# Rational Design of Highly Efficient Orange‐Red/Red Thermally Activated Delayed Fluorescence Emitters with Submicrosecond Emission Lifetimes

**DOI:** 10.1002/advs.202300808

**Published:** 2023-06-06

**Authors:** Jia‐Xuan Hu, Shanshan Jiang, Dong‐Hai Zhang, Tianxiang Zhao, Fu‐Lin Lin, Lingyi Meng, Xu‐Lin Chen, Can‐Zhong Lu

**Affiliations:** ^1^ State Key Laboratory of Structural Chemistry Fujian Institute of Research on the Structure of Matter Chinese Academy of Sciences Fuzhou Fujian 350002 China; ^2^ Xiamen Key Laboratory of Rare Earth Photoelectric Functional Materials Xiamen Institute of Rare Earth Materials Haixi Institutes Chinese Academy of Sciences Xiamen Fujian 361021 China; ^3^ School of Physical Science and Technology ShanghaiTech University Shanghai 201210 China; ^4^ Fujian Science & Technology Innovation Laboratory for Optoelectronic Information of China Fuzhou Fujian 350108 China

**Keywords:** hydrogen bonding, organic light‐emitting diodes, red emission, short emission lifetime, thermally activated delayed fluorescence

## Abstract

The development of orange‐red/red thermally activated delayed fluorescence (TADF) materials with both high emission efficiencies and short lifetimes is highly desirable for electroluminescence (EL) applications, but remains a formidable challenge owing to the strict molecular design principles. Herein, two new orange‐red/red TADF emitters, namely AC‐PCNCF3 and TAC‐PCNCF3, composed of pyridine‐3,5‐dicarbonitrile‐derived electron‐acceptor (PCNCF3) and acridine electron‐donors (AC/TAC) are developed. These emitters in doped films exhibit excellent photophysical properties, including high photoluminescence quantum yields of up to 0.91, tiny singlet‐triplet energy gaps of 0.01 eV, and ultrashort TADF lifetimes of less than 1 µs. The TADF‐organic light‐emitting diodes employing the AC‐PCNCF3 as emitter achieve orange‐red and red EL with high external quantum efficiencies of up to 25.0% and nearly 20% at doping concentrations of 5 and 40 wt%, respectively, both accompanied by well‐suppressed efficiency roll‐offs. This work provides an efficient molecular design strategy for developing high‐performance red TADF materials.

## Graphical abstract

In this work, orange‐red/red thermally activated delayed fluorescence materials achieve high photoluminescence quantum yields of up to 91% with ultrashort exciton lifetimes of less than 1 µs, demonstrating a successful balance between high efficiency and fast reverse intersystem crossing. Highly efficient organic light‐emitting diodes are realized with external quantum efficiencies of up to 25.0% and small efficiency roll‐offs.

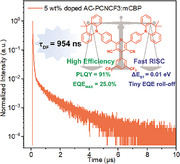

## 1 Introduction

Thermally activated delayed fluorescence (TADF) materials have drawn much academic and industrial attention in the field of organic light‐emitting diodes (OLEDs) because of their potential to realize full exciton utilization via reverse intersystem crossing (RISC).^[^ ^1^ ^]^ To date, TADF‐OLEDs displaying various emission colors with high internal quantum efficiency (IQE) of ≈100% have been reported.^[^ ^2^ ^]^ Nevertheless, high‐performance orange‐red/red TADF materials which are indispensable for constructing full‐color display and white lighting TADF‐OLEDs, are still scarce.^[^ ^3^ ^]^ Several reasons are responsible for the difficulty to develop high‐performance orange‐red/red TADF materials. First, according to the energy‐gap law, the rate of internal conversion increases exponentially with decreasing the energy gap.^[^ ^1^, ^4^ ^]^ Hence, orange‐red/red TADF molecules generally suffer from severe nonradiative deactivation of the excitons from the lowest singlet excited state (S_1_) to the ground state (S_0_), which would adversely affect the photoluminescence quantum yield (PLQY).^[^ ^4^, ^5^ ^]^ Second, TADF materials are generally featured with twisted donor‐acceptor (D‐A) molecular configurations to adequately separate the highest occupied molecular orbital (HOMO) and lowest unoccupied molecular orbital (LUMO) and obtain a small singlet‐triplet splitting (Δ*E*_ST_), which is necessary for achieving efficient RISC process at room temperature.^[^ ^6^ ^]^ However, spatially separated HOMO and LUMO can also result in a weak transition dipole moment, leading to a decreased radiative rate constant (*k*_r_) of the emissive S_1_ state and a low PLQY.^[^ ^7^ ^]^

Many molecular design strategies have been adopted for the purpose of improving the emission efficiency of orange‐red/red TADF materials. On the one hand, to suppress nonradiative decay, a prevalent strategy is using rigid and planar aromatic systems to minimize the free vibrational and rotational motions of the D‐A molecules.^[^ ^8^ ^]^ However, the rigid and planar structures often worsen self‐aggregation‐induced emission quenching in aggregative states.^[^ ^9^ ^]^ Besides, molecular *π*‐conjugation length influences the lowest locally excited state (^3^LE). A longer *π*‐conjugation length would lead to a lower‐lying ^3^LE state which may be unfavorable for the realization of small Δ*E*_ST_.^[^ ^10^ ^]^ On the other hand, to ensure efficient radiative decay, a common design of orange‐red/red TADF molecules is to compromise the Δ*E*_ST_ to a certain extent, considering the intrinsic nonradiative decays and trade‐off between *k*_r_ and Δ*E*_ST_. In such instances, according to the first‐order perturbation theory, it is hard to obtain fast RISC processes and short TADF lifetimes for organic TADF emitters which are crucial to suppress the intrinsic efficiency roll‐offs of TADF‐OLEDs under high luminance conditions.^[^ ^11^ ^]^ Therefore, it is very important but challenging to develop orange‐red/red TADF emitters with both high emission efficiencies and short exciton lifetimes. For this purpose, the molecular structures, including donor/acceptor strength, rigidity, conjugation degree, and inter‐/intramolecular interactions should be elaborately tailored.

Herein, we designed and synthesized two orange‐red/red TADF emitters, AC‐PCNCF3 and TAC‐PCNCF3, based on a pyridine‐3,5‐dicarbonitrile‐derived acceptor and two acridine donors. These orange‐red/red TADF emitters featured high emission efficiencies and ultrashort exciton lifetimes simultaneously. Our design strategy involves the unique acceptor, rigid donors as well as the significant intramolecular and intermolecular interactions. The N atoms in the 3,5‐dicyanopyridine core would form strong hydrogen bonds with the adjacent phenyl rings to confine molecular vibration and rotation and thus suppress non‐radiative decay. Trifluoromethyl substituted phenyl ring were grafted on the 3,5‐dicyanopyridine core not only to enhance the acceptor strength to realize orange‐red/red emission, but also to suppress the emission quenching caused by intermolecular *π*–*π* stacking. Unlike the commonly used donors in orange‐red/red TADF materials such as diphenylamine or triphenylamine, the acridine donors could provide larger D‐A dihedral angles and higher rigidity for the molecule, which is conducive to decreasing Δ*E*_ST_ and minimizing nonradiative deactivations at the same time. By adopting this strategy, remarkably, the doped films of AC‐PCNCF3 and TAC‐PCNCF3 (5 wt% in mCBP) exhibited high PLQYs of 0.91 and 0.60, quite small Δ*E*_ST_ of ≈0.01 eV, and very short TADF lifetimes of 954 ns and 733 ns, respectively. Eventually, the 5 wt%‐AC‐PCNCF3‐ and 5 wt%‐TAC‐PCNCF3‐doped OLEDs displayed orange‐red and red electroluminescence (EL) with high maximum external quantum efficiencies (EQEs) of 25.0% and 16.3%, respectively. For the AC‐PCNCF3‐based OLED, the efficiency roll‐off was only 11% at a high luminance of 1000 cd m^−2^. Furthermore, when the doping concentration was increased to 40%, the EQE of AC‐PCNCF3‐based OLED maintained near 20% with red‐shifted emission peaking at 600 nm and a very small efficiency roll‐off of only 8% at 1000 cd m^−2^.

## 2 Results and Discussion

### 2.1 Synthesis and Characterization

**Scheme 1:**
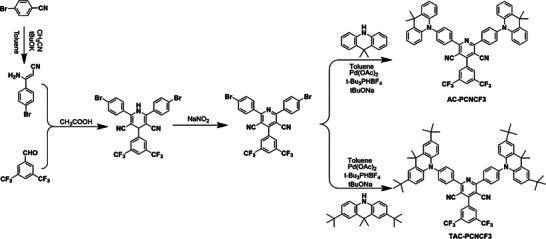
Synthetic routes of AC‐PCNCF3 and TAC‐PCNCF3.

As depicted in **Scheme** **1**, the target compounds AC‐PCNCF3 and TAC‐PCNCF3 were synthesized through a simple four‐step route containing addition‐elimination, cyclocondensation, oxidation, and Buchwald–Hartwig cross‐coupling reactions. After purification by column chromatography and vacuum sublimation, two final compounds were characterized by ^1^H‐NMR spectroscopy and elemental analysis. The synthetic procedure and characterization are detailed in the Supporting Information.

### 2.2 Theoretical Investigation

**Figure 1:**
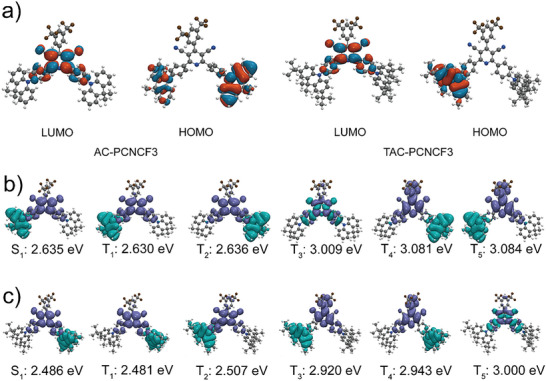
a) Frontier molecular orbital (FMO) distributions and natural transition orbitals (NTOs) for the excited states of b) AC‐PCNCF3 and c) TAC‐PCNCF3.

To predict the nature of the frontier molecular orbitals (FMOs) and excited states of AC‐PCNCF3 and TAC‐PCNCF3, density functional theory (DFT) and time‐dependent DFT (TD‐DFT) calculations were performed on both molecules at the BMK/6‐31G(d) level. The optimized ground‐state molecular geometries revealed the highly twisted structures of these molecules with large donor‐acceptor (D‐A) torsion angles of 88.7° and 89.4° for AC‐PCNCF3, and 88.1° and 89.9° for TAC‐PCNCF3 (Figure S13, Supporting Information), respectively, which would lead to effectively separated FMO distributions. As depicted in **Figure** **1**a, the HOMOs of both molecules are mainly located on the dimethylacridine units, and the LUMOs are distributed over the dicyano‐substituted pyridine core with partial distributions on the adjacent phenylene bridges. The sufficient separation of HOMOs and LUMOs contributes to the small Δ*E*_ST_ values of these two emitters. The TD‐DFT calculations revealed that the S_1_ and T_1_ states of both molecules are featured with predominant intramolecular charge transfer (ICT) transitions, with nearly equal Δ*E*_ST_ values of 0.005 eV (Table S6, Supporting Information and Figure 1b,c). Because of the symmetrical molecular structures, the excited CT states of these compounds appear in pairs degenerately (S_1_/S_2_, T_1_/T_2_, T_3_/T_4_ for TAC‐PCNCF3, T_4_/T_5_ for AC‐PCNCF3, etc.). Notably, the lowest ^3^LE states (T_3_ for AC‐PCNCF3 and T_5_ for TAC‐PCNCF3) of these molecules are predominantly localized on the acceptor units and lie much higher than the respective S_1_ and T_1_ states. The large energy gaps (0.37 eV for AC‐PCNCF3 and 0.49 eV for TAC‐PCNCF3) between the up‐lying ^3^LE states and the S_1_ states probably suggest that the ^3^LE states do not participate in the ISC and RISC processes (vide infra).

### 2.3 Single Crystal Structure Analysis

**Figure 2:**
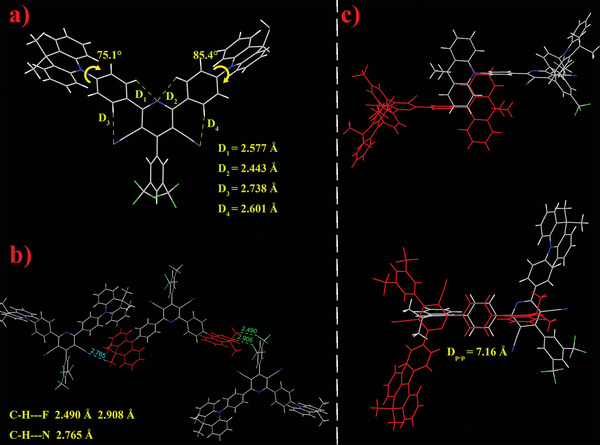
Single crystal structure and packing diagrams of AC‐PCNCF3; a) Torsion angles and intramolecular hydrogen bonds; b) Intermolecular hydrogen bonds; c) Molecular packings of AC‐PCNCF3.

To investigate the structure‐performance relationship, the single crystal of AC‐PCNCF3 was acquired by solvent diffusion method with dichloromethane and hexane (v:v = 1:2) and resolved via X‐ray crystallographic analysis. Despite deliberate attempts, we failed to obtain the single crystal of TAC‐PCNCF3, probably because of the bulky tertiary butyl groups which retard crystallization. As shown in **Figure** **2**a, the torsion angles between the two acridine donors and *π* linkers are 75.1° and 85.4°, respectively, which are expected to result in the effective separation of the HOMO and LUMO and consequently lead to a small Δ*E*_ST_.^[^ ^2a^ ^]^ Apart from using rigid and large *π*‐conjugated structure for high‐efficiency emission, which is usually not favorable for the realization of small Δ*E*_ST_ (vide supra), another effective way to increase rigidity without expanding the *π*‐conjugation length is to introduce intra‐ or intermolecular interactions.^[^ ^12^ ^]^ Noticeably, the distances between N atoms on the pyridine core and the H atoms on the phenyl linker are only 2.4–2.7 Å, indicating the formation of strong intramolecular hydrogen bonds, which would restrict the free‐rotation of phenyl rings and make the molecule more rigid and thus suppress nonradiative decay.^[^ ^12a^ ^]^ Moreover, significant intermolecular hydrogen bonding (C—H···F and C—H···N) can be observed between the adjacent molecules (Figure 2b), which further reinforces the molecular rigidity to restrict rotational and vibrational motions, thus minimizing the nonradiative relaxation.^[^ ^12b^ ^]^ Besides molecular rotation and vibration, intermolecular *π*–*π* stacking in aggregative state is another main inducement of nonradiative decay, especially for red emitters which usually need planar fragments with large *π*‐conjugation to realize low‐energy emission.^[^ ^13^ ^]^ For the investigated materials, the intermolecular *π*–*π* stacking can be effectively avoided due to the highly twisted molecular configurations. As depicted in Figure 2c, no obvious face‐to‐face *π*–*π* stacking is observed in the crystal of AC‐PCNCF3. In summary, the unique rigid and twisted molecular structures of these compounds could explain why they could realize orange‐red emission with both high PLQYs and very small Δ*E*_ST_ values. Details of the crystal data are presented in the Supporting Information (Tables S2–S4, Supporting Information). The CCDC number of AC‐PCNCF3 is 2257348.

### 2.4 Thermal and Electrochemical Properties

Thermogravimetric analysis (TGA) and differential scanning calorimetry were performed to investigate the thermal properties of AC‐PCNCF3 and TAC‐PCNCF3. The TGA results revealed their high thermal stability with decomposition temperature (*T*_d_, corresponding to the temperature of 95% weight loss) of 439 and 426 °C, respectively (Figure S5, Supporting Information). TAC‐PCNCF3 exhibited a high glass transition temperature (*T*_g_) of 224 °C, while no obvious *T*_g_ was observed for AC‐PCNCF3 in the temperature range of 50–450 K (Figure S6, Supporting Information). Such high *T*_d_ and *T*_g_ of TAC‐PCNCF3 indicate not only the ability to endure high thermal‐stress during vacuum sublimation and joule heating during the operation of OLEDs, but also the high morphological stability in doped films.

The cyclic voltammograms for the oxidation of these two compounds in dichloromethane are shown in Figure S7, Supporting Information. Interestingly, AC‐PCNCF3 and TAC‐PCNCF3 exhibited obviously different oxidation behaviors due to their different donor segments. The oxidation process of TAC‐PCNCF3 is reversible, and the cyclic voltammograms remain almost unchanged during at least 10 cycles of scans, indicating its extraordinary electrochemical stability. From the *E*_onset_ values of the first oxidative peaks, the HOMO energy levels of AC‐PCNCF3 and TAC‐PCNCF3 were determined to be −5.28 and −5.08 eV, respectively. The tert‐butyl groups of acridine make it a stronger donor and therefore TAC‐PCNCF3 has a shallower HOMO level than AC‐PCNCF3. Combined with the optical band gaps which are obtained as 2.30 and 2.14 eV from the UV–vis absorption onset values (vide infra), the LUMO levels of AC‐PCNCF3 and TAC‐PCNCF3 are calculated to be −2.98 and −2.94 eV respectively, the approximate equality of which is due to the same acceptor moiety of these two emitters. Detailed electrochemical information is summarized in Table S1, Supporting Information.

### 2.5 Photophysical Properties

**Figure 3:**
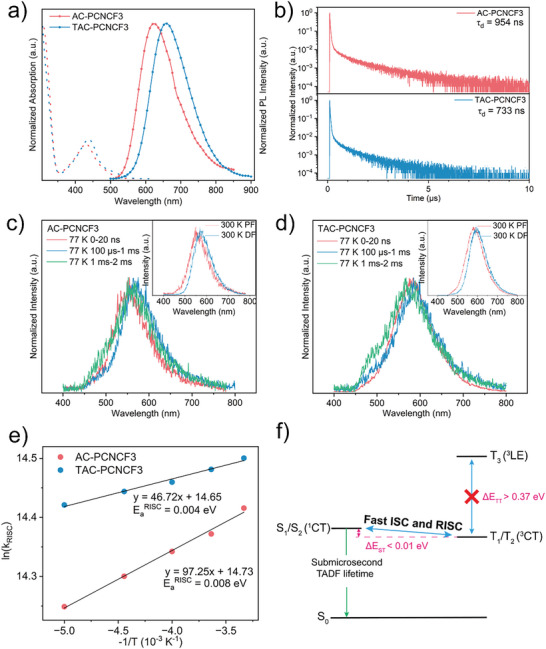
a) UV–vis absorption and fluorescence spectra recorded in dilute toluene solutions (1 × 10^−5^ m) at room temperature; b) Transient PL decay curves of AC‐PCNCF3 and TAC‐PCNCF3 in 5 wt% doped mCBP film; c) Time‐resolved transient PL spectra of AC‐PCNCF3 in 5 wt% doped mCBP film at 77 and 300 K (inset); d) Time‐resolved transient PL spectra of TAC‐PCNCF3 in 5 wt% doped mCBP film at 77 and 300 K (inset); e) Liner fit of *k*_RISC_ values at different temperatures for AC‐PCNCF3 and TAC‐PCNCF3; f) Energy‐level diagram of the excited states of AC‐PCNCF3.

Photophysical properties of these two materials were investigated in both toluene and doped films. **Figure** **3**a shows the ultraviolet‐visible (UV–vis) absorption and photoluminescence (PL) spectra of AC‐PCNCF3 and TAC‐PCNCF3 in toluene (1 × 10^−5^ m) at room temperature. The two compounds exhibit very similar absorption profiles, each consisting of a strong band below 350 nm and a relatively weak and broad band between 360 and 550 nm. The high‐energy absorption bands can be ascribed to the spin‐allowed local *π*–π* transitions, while the weak low‐energy ones are assigned to the ICT transitions from the donor units to the acceptor units. AC‐PCNCF3 and TAC‐PCNCF3 in toluene exhibit bright red (*λ*_max_ = 623 nm) and deep red emission (*λ*_max_ = 658 nm) with broad and structureless PL spectra (Figure 3a), indicating that the emissive states of both emitters possess typical CT characters. The red‐shifted PL spectrum of TAC‐PCNCF3 as compared with AC‐PCNCF3 is due to the stronger electron‐donating ability of the tert‐butyl substituted donor units. We further recorded the absorption and PL spectra of these emitters in different solvents and carried out quantitative calculation on solvatochromic effect (Figures S8,S9 and Table S5, Supporting Information).^[^ ^14^ ^]^ Both emitters exhibited a typical positive solvatochromic effect. As the solvent polarity increased, the PL spectra of these emitters were red‐shifted, together with broadened spectrum profiles, which confirms their ICT nature in emissive states.

To better understand their emission behaviors in OLEDs, the photophysical properties of AC‐PCNCF3 and TAC‐PCNCF3 were further investigated in doped films (5 wt% in mCBP host). In doped films, the steady‐state PL spectra of the AC‐PCNCF3 and TAC‐PCNCF3 peak at 572 and 603 nm, respectively, which are markedly blue‐shifted relative to those in toluene solutions (Figure S10, Supporting Information). The blue‐shifted emissions can be explained in two aspects.^[^ ^15^ ^]^ First, the solvation effect on the CT‐excited states is weakened due to the less polar environment in doped films than in toluene solutions. Second, intramolecular rotations and excited‐state distortions are effectively restricted in the more rigid environment of doped films, which would decrease the vertical transition energies from S_1_ states to S_0_ states, resulting in significantly blue‐shifted PL spectra maxima. Figure 3b shows the transient PL decay curves of these emitters in doped films at 300 K, each of which consists of typical two‐component decays, namely, a prompt fluorescence (PF) component and a delayed fluorescence (DF) component. The PF and DF lifetimes are fitted to be 17.2 and 954 ns for AC‐PCNCF3, and 19.3 and 733 ns for TAC‐PCNCF3, respectively (Figure S12, Supporting Information). The submicrosecond solid‐state TADF lifetimes are highly desirable for alleviating the intrinsic exciton‐exciton annihilation in TADF‐OLEDs. However, such short TADF lifetimes are hitherto very rare, especially for efficient orange and red TADF materials.^[^ ^16^ ^]^ As shown in Figure S11, Supporting Information, for both compounds, the intensity proportion of the delayed component decreases with the decrease of temperature, confirming their TADF characteristics. The short DF lifetimes are probably related to a fast RISC process between the S_1_ and T_1_ states. To reveal the excited state nature, the time‐resolved PL spectra of these two emitters in the doped mCBP films were recorded at 300 and 77 K. At 300 K, delayed fluorescence spectra of both emitters slightly red‐shift relative to the respective similarly‐shaped prompt fluorescence spectra (insets of Figure 3c,d). The red shift of delayed fluorescence spectra in rigid matrix can be attributed to the stabilization of S_1_ states which originate from the D‐A rotational motions after finishing excitation.^[^ ^4^, ^17^ ^]^ Figure 3c,d shows the PL spectra at each delay time at 77 K. Both emitters show typical CT‐featured prompt fluorescence spectra (recorded at 0–20 ns). And interestingly, the phosphorescence spectra recorded at 0.1–1 ms and 1–2 ms indicate a LE contribution in the T_1_ states which gradually increased with prolonging the delay time (Figure 3c,d). This phenomenon could probably be rationalized with the D‐A rotational motions (range from ns to ms) in rigid matrix.^[^ ^4^, ^17^ ^]^ After a relatively long delay time, for example, 1–2 ms, the D‐A torsion angles may decrease and thus lead to a hybrid local‐CT excited state (HLCT) excited state. According to the prompt fluorescence and phosphorescence spectra, both the Δ*E*_ST_ values of AC‐PCNCF3 and TAC‐PCNCF3 were estimated to be roughly 0.01 eV. Such small Δ*E*_ST_ would favor the realization of efficient RISC processes for both TADF emitters. The small Δ*E*_ST_ could be clarified in two aspects apart from large D‐A dihedral angles. On the one hand, the electron‐withdrawing trifluoromethyl substituents increase the acceptor strength, resulting in lower‐energy and energetically close S_1_ and T_1_ states with predominant CT characteristics. On the other hand, due to short *π*‐conjugation length, the molecules are expected to have a high lying ^3^LE state, which would result in large ^3^LE‐^3^CT energy gap and minimize the impact of ^3^LE on the small Δ*E*_ST_ (in contrast to those that have very low ^3^LE levels). At room temperature, the PLQYs (*Φ*_PL_) of AC‐PCNCF3 and TAC‐PCNCF3 in the doped mCBP films (5 wt%) are 0.91 and 0.60, respectively. According to the energy‐gap law, TAC‐PCNCF3 with lower emissive energy is more likely to suffer from severe non‐radiative decay as compared with AC‐PCNCF3, thus leading to a relatively lower PLQY. Moreover, the detrimental intermolecular interactions in doped films which could cause severer concentration quenching (vide infra) may be another reason for the lower PLQY of TAC‐PCNCF3. Combining with the intensity proportions of the two decay components in transient PL decay curves, the quantum efficiencies of prompt fluorescence (*Φ*_PF_) and delayed fluorescence (*Φ*_DF_) were calculated to be 53.0 and 37.9% for AC‐PCNCF3 and 41.7 and 18.2% for TAC‐PCNCF3, respectively (**Table** **1**). Based on the lifetime and PLQY values, the photophysical rate constants were calculated according to a reported method (See Supporting Information for details),^[^ ^18^ ^]^ and the relevant data are listed in Table 1. AC‐PCNCF3 and TAC‐PCNCF3 show radiative rate constants ($k_{r}^{S}$) of 3.04 × 10^7^ and 2.14 × 10^7^ s^−1^, nonradiative rate constants ($k_{\mathrm{nr}}^{S}$) of 3.1 × 10^6^ and 1.43 × 10^7^ s^−1^, and ISC rate constants (*k*_ISC_) of 2.4 × 10^7^ and 1.56 × 10^7^ s^−1^, respectively. Importantly, both emitters possess very fast RISC processes with rate constants (*k*_RISC_) of 1.82 × 10^6^ for AC‐PCNCF3 and 1.98 × 10^6^ s^−1^ for TAC‐PCNCF3. According to the classical Arrhenius equation, *k*_RISC_ is proportional to exp(‐*E*_a_^RISC^/*k*_B_ *T*), where *E*_a_^RISC^, *k*_B_, and *T* represent thermal activation energy of RISC process, Boltzmann constant, and temperature, respectively. From the Arrhenius plots of *k*_RISC_ (Figure 3e), *E*_a_^RISC^ values were fitted to be 8 meV for AC‐PCNCF3 and 4 meV for TAC‐PCNCF3, which are roughly in agreement with the Δ*E*_ST_ values obtained from the time‐resolved spectra and theoretical calculations. These results indicate that the higher‐lying excited states (T_3_, T_4_, T_5_, etc.) of these emitters are not directly involved as intermediate states in the RISC processes, considering that they lie much higher than the nearly degenerate S_1_/S_2_ and T_1_/T_2_ states (with energy gaps of at least 0.37 eV according to the theoretical calculations, Figure 3f). According to the El‐Sayed rule, the SOC between S_1_ and T_1_ states that have the same CT transition character is usually considered negligible. However, some recent studies indicate that the El‐Sayed rule has limitations in inhomogeneous media.^[^ ^19^ ^]^ By taking into account the contribution of rotamers in films, the statistically weighted SOC between ^1^CT and ^3^CT is not zero.^[^ ^19b^ ^]^ Moreover, it was found that the T_1_ state may have a mixed LE/CT character, and the extent of the mixing is dependent on the instantaneous molecular conformation (namely the torsion angle between donor and acceptor).^[^ ^4^, ^17^, ^20^ ^]^ This time‐dependent LE/CT character of T_1_ state may result in a time‐dependent SOC boost. In our case, the time‐resolved phosphorescence spectra of film samples (vide supra, Figure 3c,d) show a slight LE contribution in T_1_ state, which is probably caused by instantaneous D‐A rotational motions. Considering the large energy gap between ^3^LE and ^3^CT, this contribution may be very limited but ensures the statistically weighted SOC between S_1_ and T_1_ is not zero, thus opening a possible gateway for spin flip. Therefore, for the film samples, the SOC between S_1_ and T_1_ states should be actually non‐negligible. According to the first‐order perturbation theory, such SOC interactions and the very small Δ*E*_ST_ lead to the fast RISC and short fluorescence lifetimes of these emitters, even without the direct participation of intermediate ^3^LE states.

**Table 1:** Photophysical data of AC‐PCNCF3 and TAC‐PCNCF3

Compound	*λ*_PL_ ^a)^	*Φ*_PL_ ^a)^	*Φ*_PF_/*Φ*_DF_ [%]^a)^	*τ*_PF_/*τ*_DF_ [ns/ns]^a)^	*k*_RISC_ [10^6^ s^−1^]^a)^	$k_{r}^{s}$/$k_{\mathrm{nr}}^{s}$/*k*_ISC_ [10^7^ s^−1^]^b)^	*S*_1_/*T*_1_/Δ*E*_ST_ [eV]^a)^
AC‐PCNCF3	572	0.91	53.0/37.9	17.2/954	1.82	3.04/0.31/2.40	2.59/2.58/0.01
TAC‐PCNCF3	603	0.60	41.7/18.2	19.3/733	1.98	2.14/1.43/1.56	2.43/2.42/0.01

^a)^ Determined using the 5 wt% doped films in a host matrix (mCBP for both compounds) at 300 K;

^b)^ $k_{r}^{s}$, $k_{\mathrm{nr}}^{s}$, and *k*_ISC_ represent the radiative rate constant, nonradiative rate constant, and intersystem‐crossing (ISC) rate constant of the S_1_ states, respectively.

### 2.6 Electroluminescence Performance

**Figure 4:**
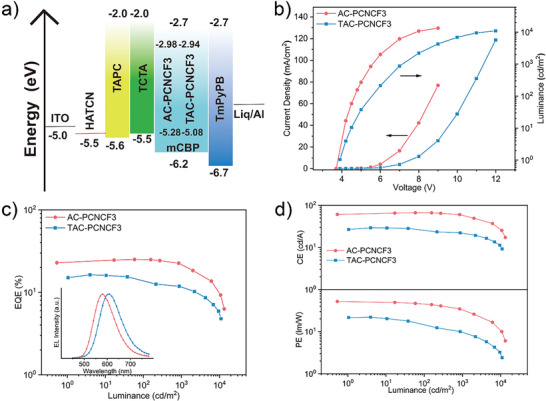
a) Energy diagram of OLEDs; b) Current density–luminance–voltage (*J*–*L*–*V*) characteristics of AC‐PCNCF3 and TAC‐PCNCF3; c) EQE versus luminance characteristics of AC‐PCNCF3 and TAC‐PCNCF3 and their EL spectra (inset) recorded at 6 V; d) PE and CE versus luminance characteristics.

To evaluate the TADF emitters in EL devices, we fabricated multilayer OLEDs by vacuum deposition with an optimized device structure of ITO/HAT‐CN (10 nm)/TAPC (30 nm)/TCTA (5 nm)/mCBP: emitters (20 nm)/TmPyPB (35 nm)/Liq (1 nm)/Al (100 nm), in which HAT‐CN, TAPC, TCTA, mCBP, TmPyPB, and Liq serve as hole‐injection layer, hole‐transporting layer, electron‐blocking layer, host, electron‐transporting layer and electron‐injecting layer, respectively. The energy‐level diagram of these devices is depicted in **Figure** **4**a. The chemical structures of the used organic materials are shown in Figure S15, Supporting Information.

We investigated the doping concentration dependence of EL performance, with emitter doping concentrations of 3, 5, 10, 15, 20, 30, 40 wt%, and 100%, as summarized in Figure S14 and Tables S7,S8, Supporting Information. A gradual spectral redshift of EL was observed when increasing the doping concentration, with emission peaks ranging from 578 to 626 nm and from 601 to 653 nm for AC‐PCNCF3‐ and TAC‐PCNCF3‐based devices respectively when going from 3 to 100 wt% (Table S7, Supporting Information). The doping concentration‐dependent EL can be ascribed to the solid solvation effect. At high concentration levels, the emissive CT states of the emitters would be stabilized by the highly polar neighboring molecules. Although the EQE_max_ of the non‐doped OLEDs employing AC‐PCNCF3 and TAC‐PCNCF3 are 7.3% and 4.4%, the EQE_max_ values of the doped OLEDs gradually increased with decreasing doping concentration (Figure S14 and Table S8, Supporting Information), reaching maximum values of 25.0% and 16.3% in the 5 wt% doped devices of AC‐PCNCF3 and TAC‐PCNCF3, respectively. Remarkably, thanks to the short exciton lifetime and fast RISC process of the TADF emitter, the AC‐PCNCF3‐based OLEDs exhibited small efficiency roll‐offs even at high doping concentration levels, for instance, a maximum EQE value of almost 20% and extremely low efficiency roll‐off of 8% at 1000 cd m^−2^ were realized at a doping concentration of 40 wt%.

We summarize and highlight the EL data of the 5 wt% doped OLEDs of AC‐PCNCF3 and TAC‐PCNCF3 in **Table** **2** and Figure 4. The AC‐PCNCF3 and TAC‐PCNCF3 doped devices turned on at 3.8 and 4.0 V, and displayed bright orange to red emission with spectra peaks of 581 and 603 nm and Commission Internationale de L'Eclairage (CIE) coordinates of (0.50, 0.49) and (0.56, 0.43), respectively. The AC‐PCNCF3‐based device showed excellent performance with a maximum EQE of 25.0%, a maximum power efficiency (PE) of 51.7 lm W^−1^, a maximum current efficiency (CE) of 66.7 cd A^−1^, and a maximum luminance of 13 280 cd m^−2^, while the TAC‐PCNCF3‐based device only achieved a maximum EQE of 16.3% probably due to the much lower PLQY. Especially, the EQE values of the 5 wt% AC‐PCNCF3‐doped device remained at a high level of 24.9% and 22.2% at a practical luminance of 100 and 1000 cd m^−2^, respectively, which are markedly superior to those of the counterpart device based on TAC‐PCNCF3. Considering that these two TADF emitters have similar delayed fluorescence lifetimes and *k*_RISC_, the relatively larger efficiency roll‐offs of TAC‐PCNCF3‐doped devices may be attributed to the detrimental intermolecular interactions in the emitting layer, which can be reasonably inferred from the severer concentration quenching compared with AC‐PCNCF3‐doped devices.

**Table 2:** Summary of electroluminescence data of AC‐PCNCF3 and TAC‐PCNCF3

Compound	*V*_on_ [V]^a)^	*L*_max_ [cd m^−2^]^b)^	CE [cd A^−1^]^c)^	PE [lm W^−1^]^c)^	EQE [%]^c)^	*λ*_EL_ [nm]^d)^	CIE (*x*,*y*)^e)^
AC‐PCNCF3	3.8	13 280	66.7/66.6/57.9	51.7/45.2/32.9	25.0/24.9/22.2	581	(0.50,0.49)
TAC‐PCNCF3	4.0	10 920	29.3/25.6/21.8	21.9/14.7/9.6	16.3/13.8/11.6	603	(0.56,0.43)

^a)^ The Turn‐on voltage at luminance of 1 cd m^−2^;

^b)^ The maximum luminescence value;

^c)^ The efficiency values of maximum/at 100 cd m^−2^/at 1000 cd m^−2^;

^d)^ The peak of the EL spectra at 6 V;

^e)^ CIE coordinates measured at 6 V.

## 3 Conclusion

In summary, two orange‐red TADF emitters AC‐PCNCF3 and TAC‐PCNCF3 comprised of pyridine‐3,5‐dicarbonitrile‐derived electron‐acceptor and acridine electron‐donors have been synthesized through facile synthetic routes. By rational design, both compounds in doped films exhibit very small Δ*E*_ST_ values of 0.01 eV, which facilitates efficient RISC process of excitons from the T_1_ to the S_1_ state, leading to ultrashort delayed fluorescence lifetimes of less than 1 µs and fast RISC rates of 1.82 × 10^6^ and 1.98 × 10^6^ s^−1^ for AC‐PCNCF3 and TAC‐PCNCF3, respectively. Remarkably, AC‐PCNCF3 also shows a high PLQY of 0.91, demonstrating a delicate balance between fast RISC and high emission efficiency. The AC‐PCNCF3‐based OLEDs achieved orange‐red/red electroluminescence emission with high EQEs of up to 25.0% and nearly 20% at doping concentrations of 5 wt% and 40 wt%, both accompanied by well‐suppressed efficiency roll‐offs of only 11% and 8%, respectively. This work demonstrates an effective way to design red TADF materials with high efficiency and low efficiency roll‐offs.

## Conflict of Interest

The authors declare no conflict of interest.

## Supporting information

Supporting Information

## Data Availability

The data that support the findings of this study are available from the corresponding author upon reasonable request.
