# Supplementary material for: Rational Design of Highly Efficient Orange‐Red/Red Thermally Activated Delayed Fluorescence Emitters with Submicrosecond Emission Lifetimes
Source: Adv Sci (Weinh). 2023 Jun 6;10(23):2300808. doi: 10.1002/advs.202300808 (PMC10427351; doi:10.1002/advs.202300808)
Supplement: Supplementary file 1 — Supporting Information [file ADVS-10-2300808-s001.pdf]

ADVANCED SCIENCE Open Access

Supporting Information

for *Adv. Sci.*, DOI 10.1002/advs.202300808

Rational Design of Highly Efficient Orange-Red/Red Thermally Activated Delayed Fluorescence Emitters with Submicrosecond Emission Lifetimes

Jia-Xuan Hu, Shanshan Jiang, Dong-Hai Zhang, Tianxiang Zhao, Fu-Lin Lin, Lingyi Meng, Xu-Lin Chen and Can-Zhong Lu**

Supporting Information

Rational Design of Highly Efficient Orange-Red/Red Thermally Activated Delayed Fluorescence Emitters with Submicrosecond Emission Lifetimes

Jia-Xuan Hu, Shanshan Jiang, Dong-Hai Zhang, Tianxiang Zhao, Fu-Lin Lin, Lingyi Meng, Xu-Lin Chen, Can-Zhong Lu**

Table of Contents

1. General Information

2. Synthesis of Materials

3. NMR Spectra

4. Thermal Properties

5. Electrochemical Properties

6. X-ray Crystallographic Analysis

7. Photophysical Properties

8. Theoretical Calculations

9. Device Fabrication

10. Reference

1. General Information

All the chemicals and reagents were purchased from commercial sources and used as received without further purification. All reactions were taken under an N_2 atmosphere using standard Schlenk techniques. ^1H NMR and ^{13}C NMR spectra were measured on a Bruker Avance III 500 MHz NMR spectrometer. Elemental analyses were taken with an Elementar Vario EL Cube elemental analyzer. Single crystal X-ray diffraction data were collected at 200K on a Bruker–D8 VENTRUE diffractometer with X-ray source of Mo Kα. Thermogravimetry-Differential Scanning Calorimeter analysis (TG-DSC) was performed by METTLER-TOLEDO TGA/DSC under nitrogen atmosphere at a heating rate of 10K/min. Cyclic voltammetry (CV) was carried out by CHI840D electrochemical analyzer. UV-vis absorption spectra were recorded with an Agilent Cary 5000 UV-Vis spectrophotometer under ambient conditions. Fluorescence quantum yields were measured using FLS-1000 spectrofluorometer equipped with an integrating sphere. Steady-state PL spectra were recorded on Edinburgh FLS980 using a xenon lamp as a light source. The transient PL decay curves of doped film samples were recorded on Edinburgh FLS980 in time-correlated single-photon counting mode with a NT242-1K OPO laser as an excitation source. Time-resolved phosphorescence spectra and Time-resolved fluorescence spectra were recorded on an Edinburgh LP980 spectrophotometer with a NT242-1K OPO laser excitation source at 77 K cooling by liquid nitrogen.

2. Synthesis of Materials

3-amino-3-(4-bromophenyl)acrylonitrile

The synthesis process was referred to the reported literature.[1]

4-(3,5-bis(trifluoromethyl)phenyl)-2,6-bis(4-bromophenyl)pyridine-3,5-dicarbonitrile (Br-PCNCF3)

3-amino-3-(4-bromophenyl)acrylonitrile (4.46 g, 20 mmol), 3,5-bis(trifluoromethyl)benzaldehyde (2.42 g, 10 mmol), acetic acid (30 ml) were added into a round-bottom flask (200 mL). Then the mixture was refluxed at 120°C for 1h under a nitrogen atmosphere. The intermediate 4-[3,5-bis(trifluoromethyl)phenyl]-2,6-bis(4-bromophenyl)-1,4-dihydropyridine-3,5-dicarbonitrile was then precipitated as white solid. ^{1}H NMR (500 MHz, $CDCl_3$) δ 7.96 (s, 1H), δ 7.90 (s, 2H), δ 7.71-7.69 (dt, 4H), δ 7.51-7.48 (dt, 4H), δ 6.47 (s, 1H), δ 4.85 (s, 1H)

Then, sodium nitrite (6.9 g, 100 mmol) was added slowly at 70 °C. The mixture was stirred for another 1 h at the same temperature. After cooled down to room temperature, the mixture was then diluted with 500 mL ice-water mixture and neutralized with ammonia. The precipitate was filtered off and washed with water to afford Br-PCNCF3 as white powder (5.3 g, 81%). ^{1}H NMR (500 MHz, $CDCl_3$) δ 8.19 (s, 1H), δ 8.11 (s, 2H), δ 8.01-7.98 (dt, 4H), δ 7.78-7.75 (dt, 4H)

2,7-di-tert-butyl-9,9-dimethyl-9,10-dihydroacridine (TAC)

The synthesis process was referred to the reported literature.[2]

4-(3,5-bis(trifluoromethyl)phenyl)-2,6-bis(4-(9,9-dimethylacridin-10(9H)-yl)phenyl)pyridine-3,5-dicarbonitrile (AC-PCNCF3)

Br-PCNCF3 (1.95 g, 3 mmol), 9,9-dimethyl-9,10-dihydroacridine (1.38 g, 6.6 mmol), sodium tert-butoxide (672 mg, 7 mmol), tri-tert-butylphosphonium tetrafluoroborate (87 mg, 0.3 mmol), palladium (II) acetate (33 mg, 0.12 mmol) and toluene (30 ml) were refluxed under argon for 24 h. After cooled, the mixture was extracted with brine and ethyl acetate, and dried over anhydrous Na_2SO_4. After removal of the solvent, the residue was purified by column chromatography on silica gel using ethyl acetate/petroleum ether (1:20 by vol.) as the eluent to give an orange powder (2.4 g, 88%). ^{1}H NMR (500 MHz, $CDCl_3$) δ 8.45-8.42 (dt, 4H), δ 8.23 (s, 1H), δ 8.19 (s, 2H), δ 7.65-7.63 (dt, 4H), δ 7.52-7.51 (dd, 4H), δ 7.07-7.03 (td, 4H), δ 7.03-6.99 (td, 4H), δ 6.45-6.43 (dd, 4H), δ 1.73 (s, 12H) Anal. Calcd for $C_{57}H_{39}F_6N_5$: C, 75.4; H, 4.33; F, 12.55; N, 7.71. Found: C, 76.51; H, 4.45; N, 7.92.

4-(3,5-bis(trifluoromethyl)phenyl)-2,6-bis(4-(2,7-di-tert-butyl-9,9-dimethylacridin-10(9H)-yl)phenyl)pyridine-3,5-dicarbonitrile (TAC-PCNCF3)

This compound was prepared by the same procedure with AC-PCNCF3 excepting using the 2,7-di-tert-butyl-9,9-dimethyl-9,10-dihydroacridine (2.12 g, 6.6 mmol). The compound is also orange solid (2.5 g, 74%). ^{1}H NMR (500 MHz, $CDCl_3$) δ 8.40 – 8.38 (dt, 4H), δ 8.21 (s, 1H), δ 8.18 (s, 2H), δ 7.63-7.60 (dt, 4H), δ 7.52 (d, 4H), δ 7.09-7.06 (dd, 4H), δ 6.45-6.43 (d, 4H), δ 1.74 (s, 12H), δ 1.34 (s, 36H) Anal. Calcd for $C_{73}H_{71}F_6N_5$: C, 77.43; H, 6.32; F, 10.07; N, 6.18. Found: C, 78.51; H, 6.30; N, 6.34.

3. NMR spectra

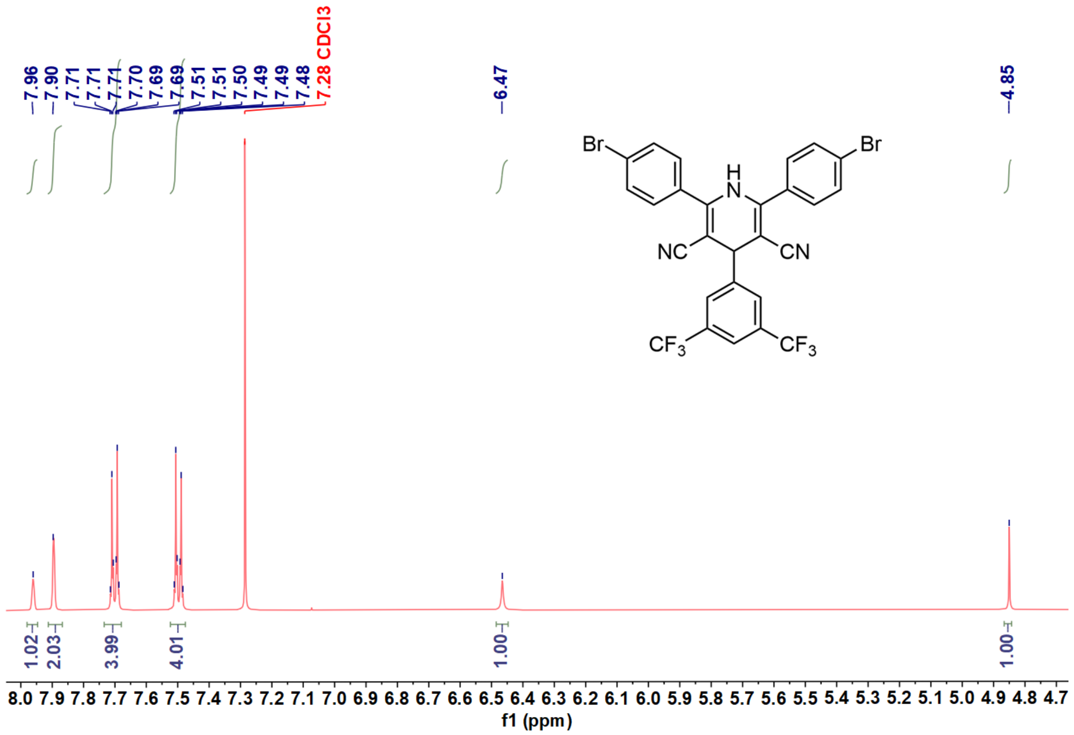

Figure S1. ^{1}H-NMR spectrum of 4-[3,5-bis(trifluoromethyl)phenyl]-2,6-bis(4-bromophenyl)-1,4-dihydropyridine-3,5-dicarbonitrile in $CDCl_3$.

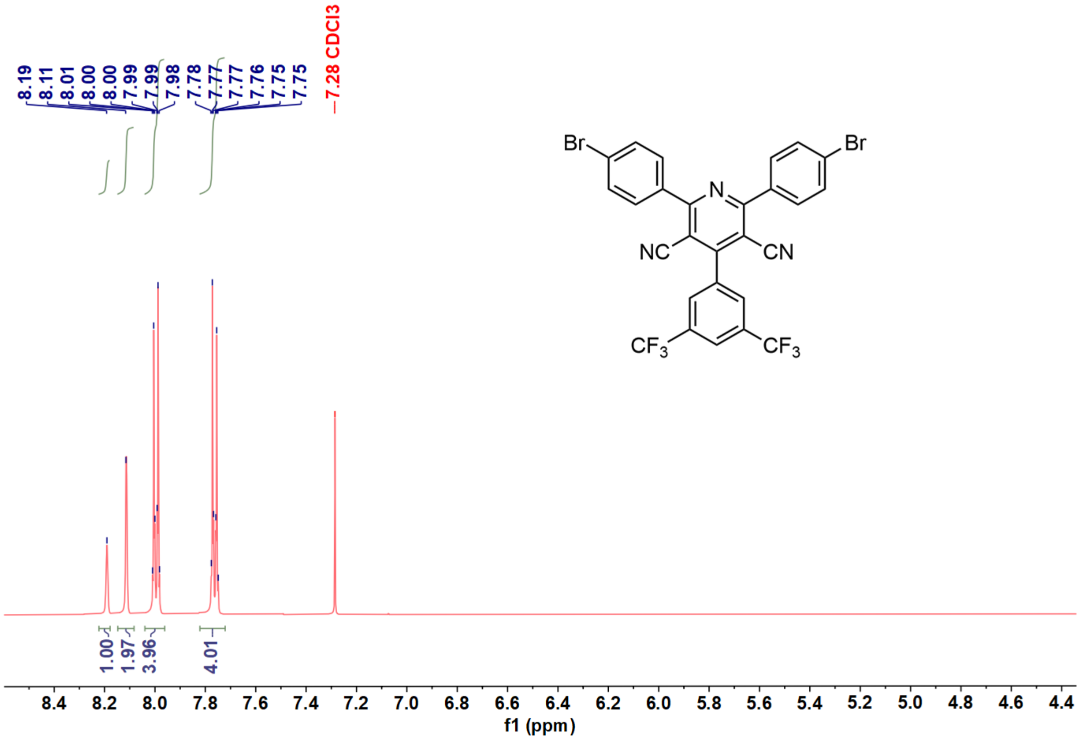

Figure S2. ^{1}H-NMR spectrum of Br-PCNCF3 in $CDCl_3$.

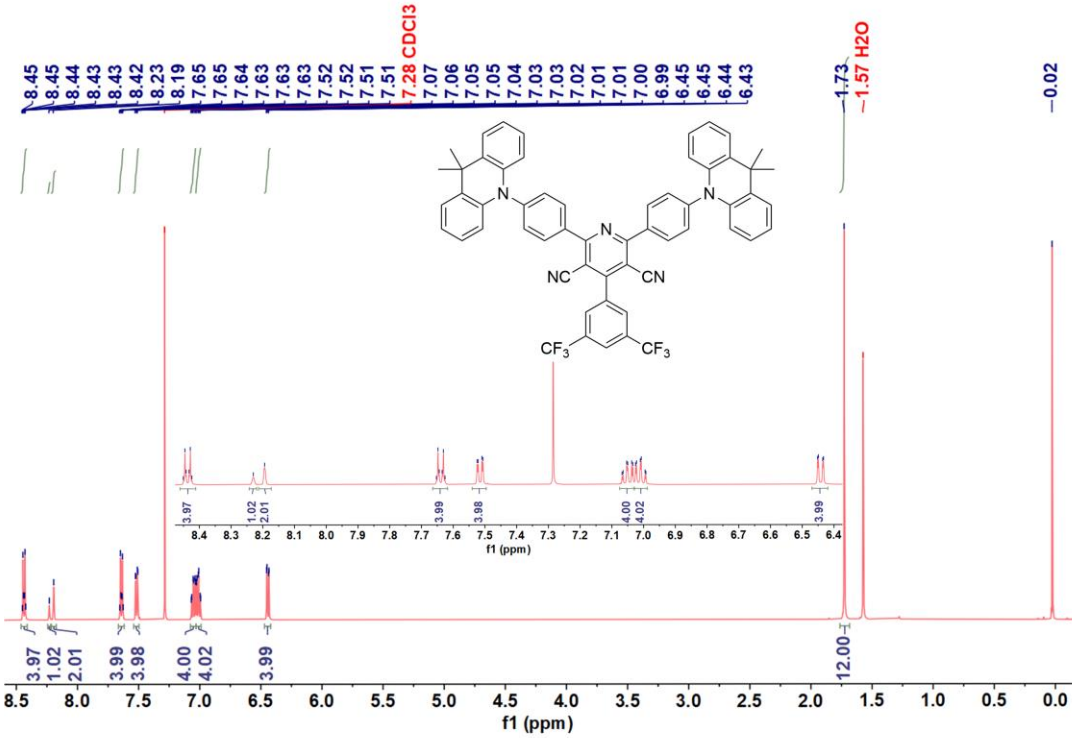

Figure S3. ^{1}H-NMR spectrum of AC-PCNCF3 in $CDCl_3$.

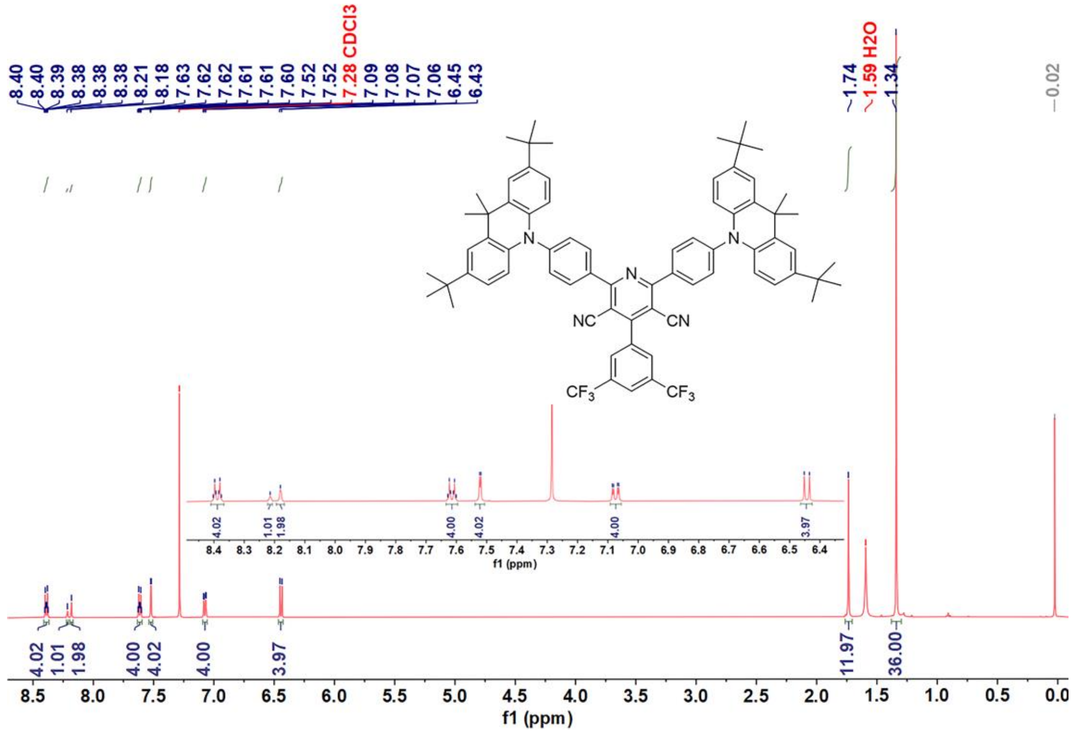

Figure S4. ^{1}H-NMR spectrum of TAC-PCNCF3 in $CDCl_3$.

4. Thermal Properties

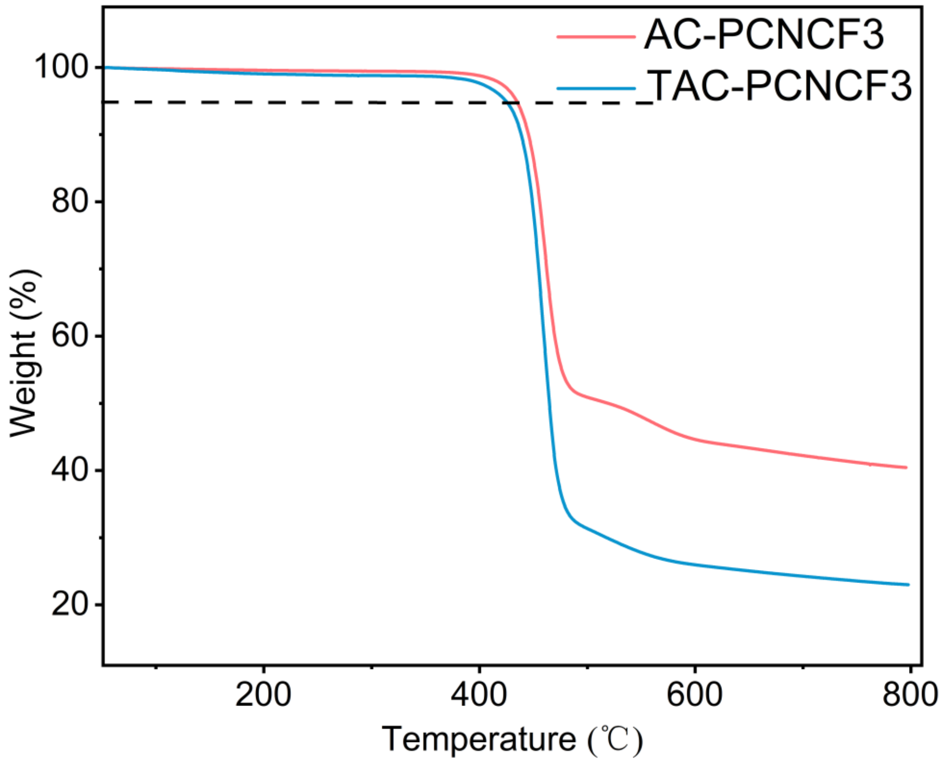

Figure S5. Thermogravimetric analysis curves of AC-PCNCF3, TAC-PCNCF3.

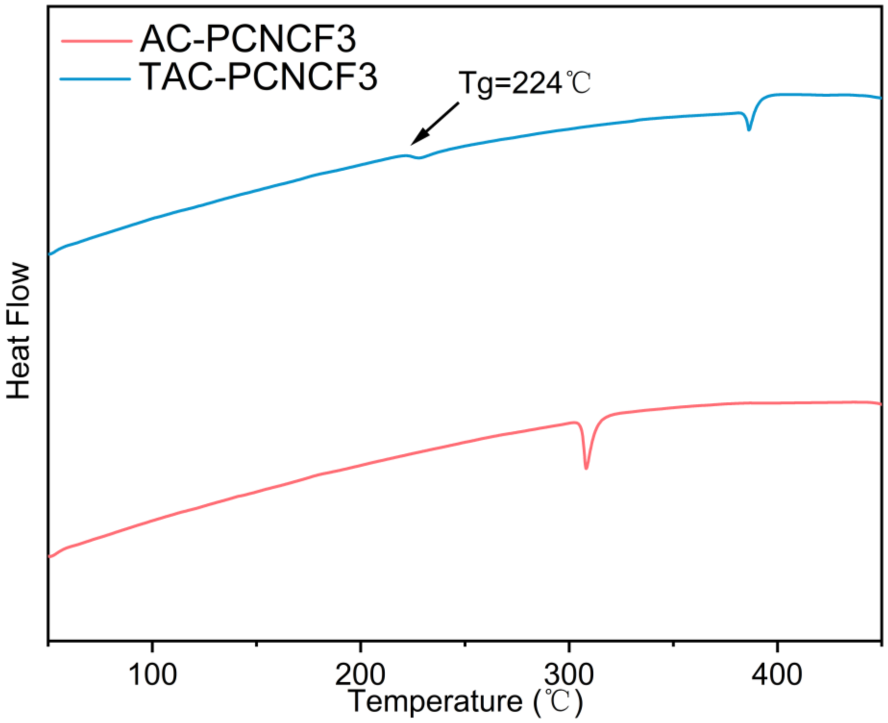

Figure S6. DSC analysis curves of AC-PCNCF3, TAC-PCNCF3.

5. Electrochemical Properties

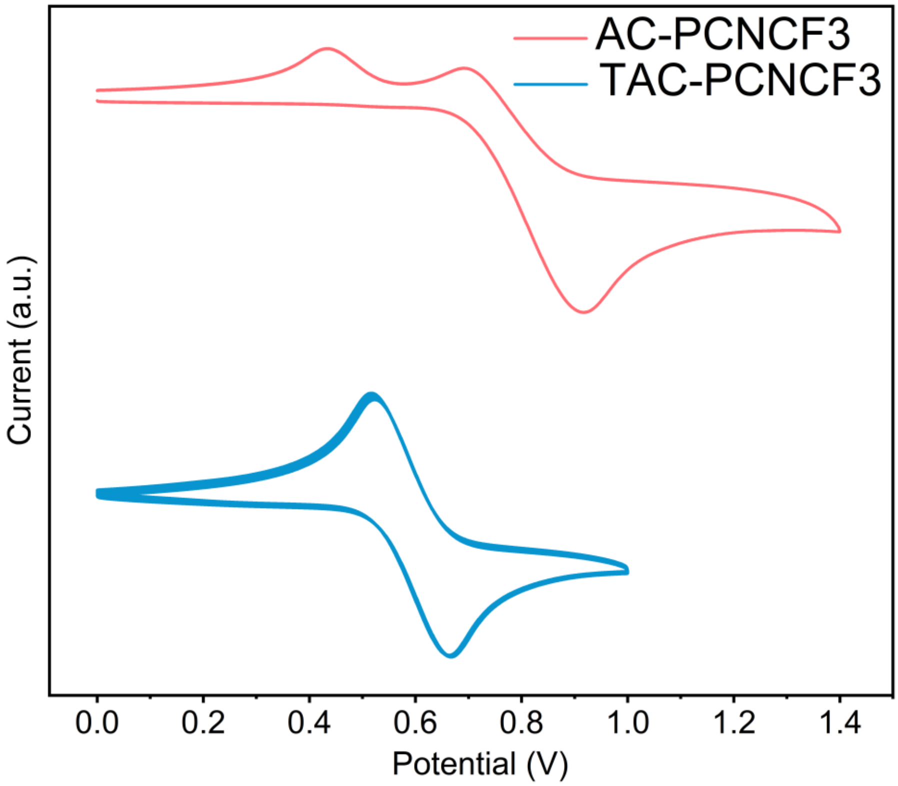

Figure S7. Cyclic Voltammograms of AC-PCNCF3 and TAC-PCNCF3.

The CV measurements were carried out in anhydrous and nitrogen-saturated dichloromethane (DCM) solutions with 0.1 M n-Bu_4NPF_6 and 1.0 mM investigated compounds. Using glassy carbon electrode as working electrode, platinum wire as auxiliary electrode, porous glass wick $Ag/AgNO_3$ as reference electrode and ferrocene/ferrocenium as the internal standard. The HOMO energy level was calculated from the onset potential of oxidation by cyclic voltammetry.

[HOMO = - (4.8- $E_{1/2(Fc/Fc+)}$ + E_{onset})]

The LUMO energy level was determined from the difference between the HOMO levels and optical band gap (E_g) estimated from the onset of the UV-Vis absorption band.

$E_g = 1241 / \lambda_{onset}$ [LUMO = HOMO + E_g]

Table S1. HOMO, LUMO and energy gap levels of AC-PCNCF3 and TAC-PCNCF3

Compound	HOMO (eV) [a)]	LUMO (eV)[c)]	E_g (eV)[b)]
AC-PCNCF3	-5.28	-2.98	2.3
TAC-PCNCF3	-5.08	-2.94	2.14

[a)] HOMO energy levels were determined from CV data; [b)] Optical band gap energies (E_g) were determined from the onsets of the corresponding UV–vis absorption spectra; [c)] LUMO energy levels were calculated from HOMO and E_g.

6. X-ray Crystallographic Analysis

Table S2. Crystal data and structure refinements for AC-PCNCF3

Compounds	AC-PCNCF3
Empirical formula	$C_{57}H_{39}F_6N_5$
Formula weight	907.93
Temperature/K	200.0
Crystal system	triclinic
Space group	P-1
a/Å	8.0130(4)
b/Å	15.0465(7)
c/Å	19.2531(10)
α/°	75.661(2)
β/°	85.799(2)
γ/°	84.677(2)
Volume/Å^3	2236.24(19)
Z	2
ρ_{calc}g/cm^3	1.3483
μ/mm^{-1}	0.10
F(000)	940.0
Crystal size/mm^3	0.200 × 0.005 × 0.005
Radiation	MoKα (λ = 0.71073)
2Θ range for data collection/°	3.95 to 55.14

Index ranges	-10 ≤ h ≤ 10, -19 ≤ k ≤ 19, -25 ≤ l ≤ 25
Reflections collected	95594
Independent reflections	10307 [R_{int} = 0.1126, R_{sigma} = 0.0563]
Data/restraints/parameters	10307/0/618
Goodness-of-fit on F^2	1.0200
Final R indexes [I>=2σ (I)]	R_1 = 0.0506, wR_2 = 0.1046
Final R indexes [all data]	R_1 = 0.1045, wR_2 = 0.1337
Largest diff. peak/hole / e Å^{-3}	0.26/-0.31

Table S3. Bond Lengths for AC-PCNCF3.

Atom	Atom	Length/Å	Atom	Atom	Length/Å
F1	C52	1.326(3)	C16	C38	1.522(3)
F2	C52	1.328(3)	C17	C18	1.379(3)
F3	C52	1.325(3)	C18	C41	1.380(3)
F4	C54	1.334(2)	C19	C20	1.377(3)
F5	C54	1.336(3)	C19	C27	1.379(4)
F6	C54	1.351(3)	C20	C21	1.372(3)
N1	C4	1.411(3)	C21	C22	1.399(3)
N1	C5	1.430(3)	C22	C26	1.397(3)
N1	C22	1.402(3)	C23	C24	1.375(3)
N2	C9	1.337(2)	C25	C26	1.520(3)

N2	C10	1.344(2)	C25	C28	1.546(3)
N3	C14	1.434(2)	C25	C29	1.539(3)
N3	C15	1.402(3)	C26	C27	1.396(3)
N3	C32	1.403(2)	C30	C31	1.381(3)
N4	C56	1.145(3)	C32	C33	1.396(3)
N5	C57	1.146(3)	C32	C37	1.399(3)
C1	C2	1.378(4)	C33	C34	1.388(3)
C1	C23	1.371(4)	C33	C38	1.526 (3)
C2	C3	1.395(3)	C34	C35	1.378(3)
C3	C4	1.394(3)	C35	C36	1.376(3)
C3	C25	1.519(3)	C36	C37	1.371(3)
C4	C24	1.398(3)	C38	C39	1.540(3)
C5	C6	1.384(3)	C38	C40	1.541(3)
C5	C30	1.380(3)	C41	C42	1.374(3)
C6	C7	1.381(3)	C43	C44	1.383(3)
C7	C8	1.394(3)	C45	C46	1.399(3)
C8	C9	1.486(3)	C45	C57	1.437(3)
C8	C31	1.393(3)	C46	C47	1.493(3)
C9	C55	1.413(3)	C46	C55	1.394(3)
C10	C11	1.484(3)	C47	C48	1.387 (3)
C10	C45	1.411(3)	C47	C53	1.390(3)
C11	C12	1.389(3)	C48	C49	1.394(3)

C11	C44	1.396(3)	C49	C50	1.384(3)
C12	C13	1.380(3)	C49	C54	1.483(3)
C13	C14	1.375(3)	C50	C51	1.383(3)
C14	C43	1.389(3)	C51	C52	1.491(3)
C15	C16	1.398(3)	C51	C53	1.384(3)
C15	C42	1.401(3)	C55	C56	1.438(3)
C16	C17	1.389(3)			

Table S4. Bond angles for AC-PCNCF3

Atom	Atom	Atom	Angle/°	Atom	Atom	Atom	Angle/°
C4	N1	C5	119.10(17)	C3	C25	C28	107.4(2)
C22	N1	C4	121.83(17)	C3	C25	C29	109.94(19)
C22	N1	C5	118.99(17)	C26	C25	C28	107.15(18)
C9	N2	C10	121.24(17)	C26	C25	C29	111.1(2)
C15	N3	C14	119.88(16)	C29	C25	C28	109.9(2)
C15	N3	C32	121.58(16)	C22	C26	C25	121.89(19)
C32	N3	C14	117.99(16)	C27	C26	C22	117.2(2)
C23	C1	C2	118.9(2)	C27	C26	C25	120.6(2)
C1	C2	C3	122.8(2)	C19	C27	C26	122.6(2)
C2	C3	C25	120.1(2)	C5	C30	C31	120.5(2)
C4	C3	C2	117.3(2)	C30	C31	C8	120.27(19)
C4	C3	C25	122.42(19)	C33	C32	N3	120.05(18)

C3	C4	N1	119.85(19)	C33	C32	C37	120.13(18)
C3	C4	C24	120.0(2)	C37	C32	N3	119.82(18)
C24	C4	N1	120.20(19)	C32	C33	C38	121.64(17)
C6	C5	N1	121.52(19)	C34	C33	C32	117.44(19)
C30	C5	N1	119.03(19)	C34	C33	C38	120.78(18)
C30	C5	C6	119.45(19)	C35	C34	C33	122.3(2)
C7	C6	C5	120.5(2)	C36	C35	C34	119.5(2)
C6	C7	C8	120.2(2)	C37	C36	C35	119.9(2)
C7	C8	C9	119.26(18)	C36	C37	C32	120.6(2)
C31	C8	C7	118.94(19)	C16	C38	C33	110.80(16)
C31	C8	C9	121.81(18)	C16	C38	C39	107.86(17)
N2	C9	C8	116.16(17)	C16	C38	C40	110.78(18)
N2	C9	C55	121.03(18)	C33	C38	C39	107.72(18)
C55	C9	C8	122.75(18)	C33	C38	C40	110.44(18)
N2	C10	C11	115.04(17)	C39	C38	C40	109.13(19)
N2	C10	C45	120.26(18)	C42	C41	C18	120.1(2)
C45	C10	C11	124.66(18)	C41	C42	C15	120.5(2)
C12	C11	C10	122.94(19)	C44	C43	C14	120.2(2)
C12	C11	C44	118.47(19)	C43	C44	C11	120.4(2)
C44	C11	C10	118.59(18)	C10	C45	C57	123.35(18)
C13	C12	C11	120.8(2)	C46	C45	C10	119.81(18)
C14	C13	C12	120.5(2)	C46	C45	C57	116.77(18)

C13	C14	N3	119.23(19)	C45	C46	C47	120.67(18)
C13	C14	C43	119.49(19)	C55	C46	C45	118.39(18)
C43	C14	N3	121.2(2)	C55	C46	C47	120.95(18)
C16	C15	N3	120.13(17)	C48	C47	C46	120.06(18)
C16	C15	C42	120.02(19)	C48	C47	C53	119.77(19)
C42	C15	N3	119.85(18)	C53	C47	C46	120.16(19)
C15	C16	C38	121.66(18)	C47	C48	C49	120.0(2)
C17	C16	C15	117.70(19)	C48	C49	C54	119.78(19)
C17	C16	C38	120.45(18)	C50	C49	C48	120.1(2)
C18	C17	C16	122.3(2)	C50	C49	C54	119.97(19)
C17	C18	C41	119.3(2)	C51	C50	C49	119.6(2)
C20	C19	C27	119.1(2)	C50	C51	C52	119.6(2)
C21	C20	C19	120.2(2)	C50	C51	C53	120.7(2)
C20	C21	C22	120.7(2)	C53	C51	C52	119.7(2)
C21	C22	N1	119.64(19)	C51	C53	C47	119.8(2)
C26	C22	N1	120.24(19)	C9	C55	C56	122.31(18)
C26	C22	C21	120.1(2)	C46	C55	C9	119.24(18)
C1	C23	C24	120.3(2)	C46	C55	C56	118.40(18)
C23	C24	C4	120.7(2)	N4	C56	C55	177.6(3)
C3	C25	C26	111.27(17)	N5	C57	C45	177.0(2)

7. Photophysical Properties

This section is refered to the literature.[3]

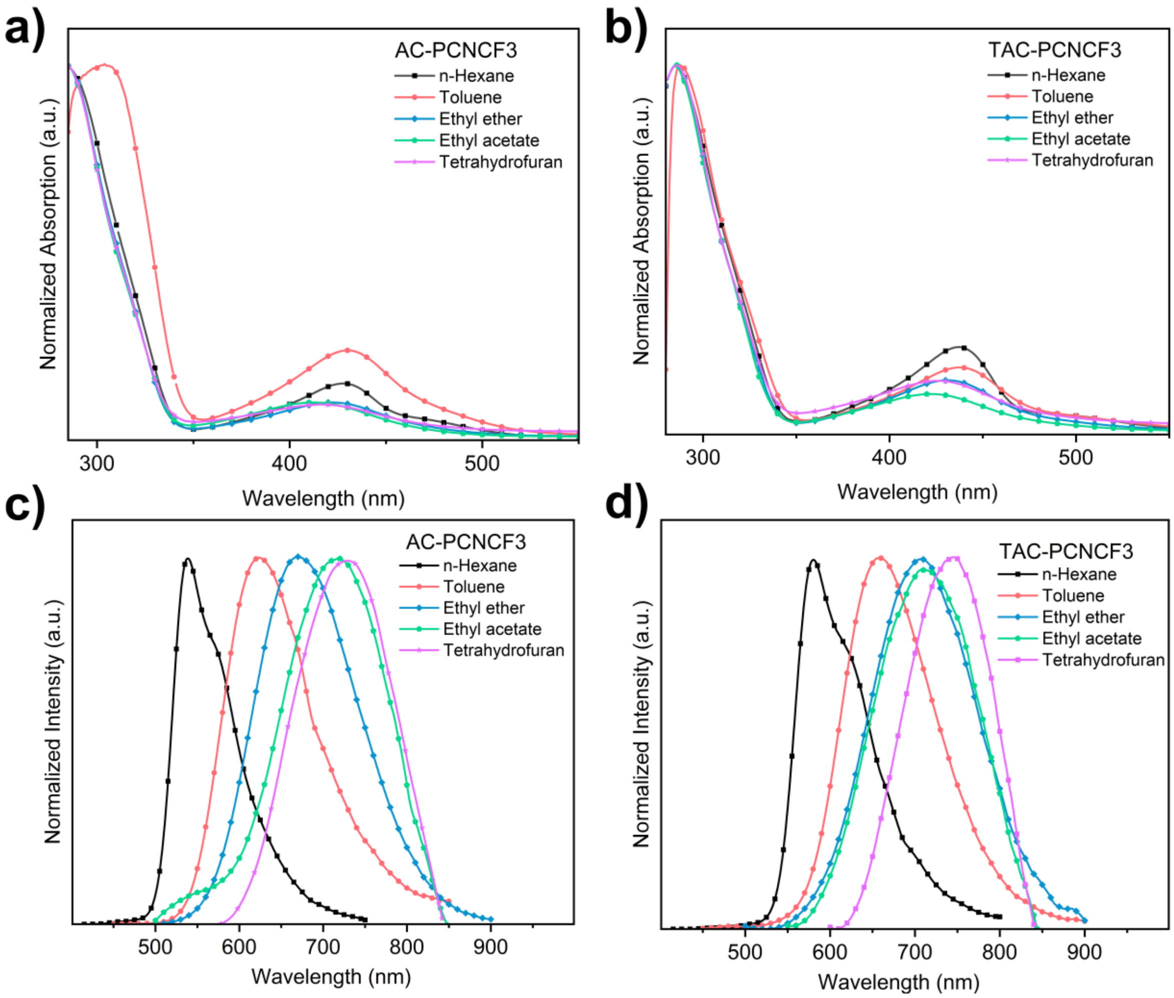

Figure S8. Absorption spectra of a) AC-PCNCF3 and b) TAC-PCNCF3 measured at room temperature in various solvents, and fluorescence spectra of c) AC-PCNCF3 and d) TAC-PCNCF3 at room temperature measured in various solvents.

Lippert–Mataga analysis for the solvatochromic effect. Both compounds exhibit clear solvatochromic shift of the fluorescence spectra from green to yellow emissions 539 and 580 nm in hexane to near infrared emissions 730 and 745 nm in Tetrahydrofuran. The dependence of the Stokes shift between the absorbance and PL spectra in each polar solvent can be

constructed by the Lippert–Mataga equation:

$$\text{Stokes shift}(\mathbf{va} - \mathbf{vp}) = \left[\frac{2(\mu_e - \mu_g)}{hca^3}\right] f(\varepsilon, n) + const$$

In the Lippert-Mataga formalism, ε and n denote the dielectric constant and the refractive index of solvent, respectively. h is Planck's constant, c is the speed of light. a is Onsager cavity radius. μ_g and μ_e are dipole moments in the ground state and the excited state, respectively. The orientation polarizability $f(\varepsilon,n)$ is defined as follows:

$$f(\varepsilon, n) = \left[\frac{\varepsilon - 1}{2\varepsilon + 1}\right] - \left[\frac{n^2 - 1}{2n^2 + 1}\right]$$

Table S5. The UV-vis, PL data and Stokes shift for AC-PCNCF3 and TAC-PCNCF3 in different solvents.

Solvent [1.0×10^{-5} M]	ε[a)]	n[a)]	f(ε,n)[a)]	Abs_{max} [nm][b)]	PL_{max} [nm][b)]	$\nu_a - \nu_p$ [cm^{-1}][b)]	Abs_{max} [nm][c)]	PL_{max} [nm][c)]	$\nu_a - \nu_p$ [cm^{-1}][c)]
n-Hexane	1.9	1.375	0.0013	428	539	4811	437	580	5642
Toluene	2.4	1.497	0.0132	430	623	7204	438	658	7633
Ethyl Ether	4.3	1.352	0.1667	422	669	8749	431	708	9078
Ethyl acetate	6.4	1.372	0.2060	412	719	10364	422	708	9572
Tetrahydrofuran	7.6	1.407	0.2096	412	730	10573	425	745	10107

[a)] The ε and n values are obtained from http://www.stenutz.eu/chem/solv23.php; [b)] Absorption maximum (Abs_{max}), fluorescence maximum (PL_{max}) and Stokes shift ($\nu_a - \nu_p$) of AC-PCNCF3 in different solvents; [c)] Absorption maximum (Abs_{max}), fluorescence maximum (PL_{max}) and Stokes shift ($\nu_a - \nu_p$) of TAC-PCNCF3 in different solvents.

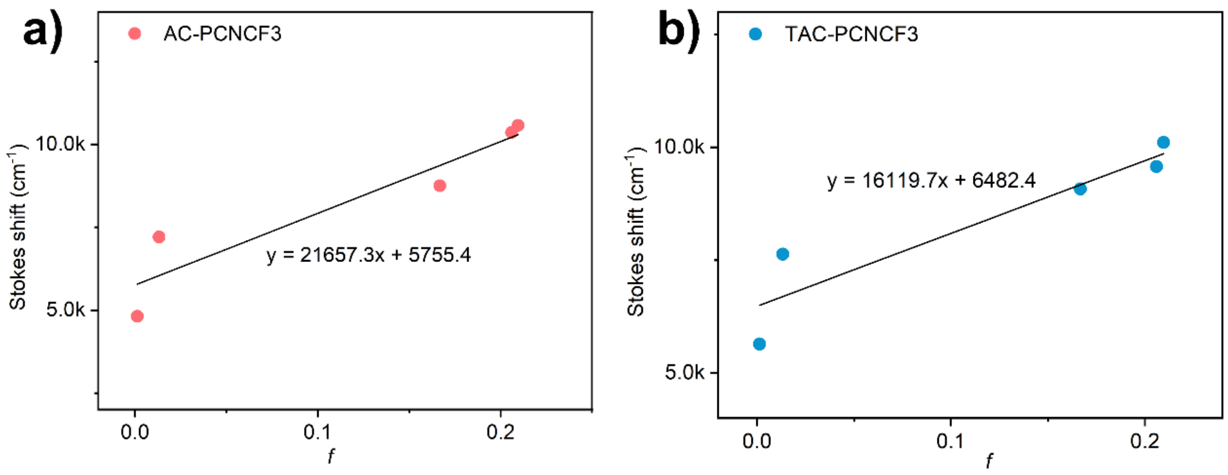

Figure S9. Lippert-Mataga plots for a) AC-PCNCF3 and b) TAC-PCNCF3.

Based on the Lippert-Mataga equation calculated and performed above, the $\Delta\mu$ (μ_e - μ_g) was estimated via the linear slope from the Lippert-Mataga plots. For AC-PCNCF3 and TAC-PCNCF3, the slopes are 21657.3 cm^{-1} and 16119.7 cm^{-1}, respectively.

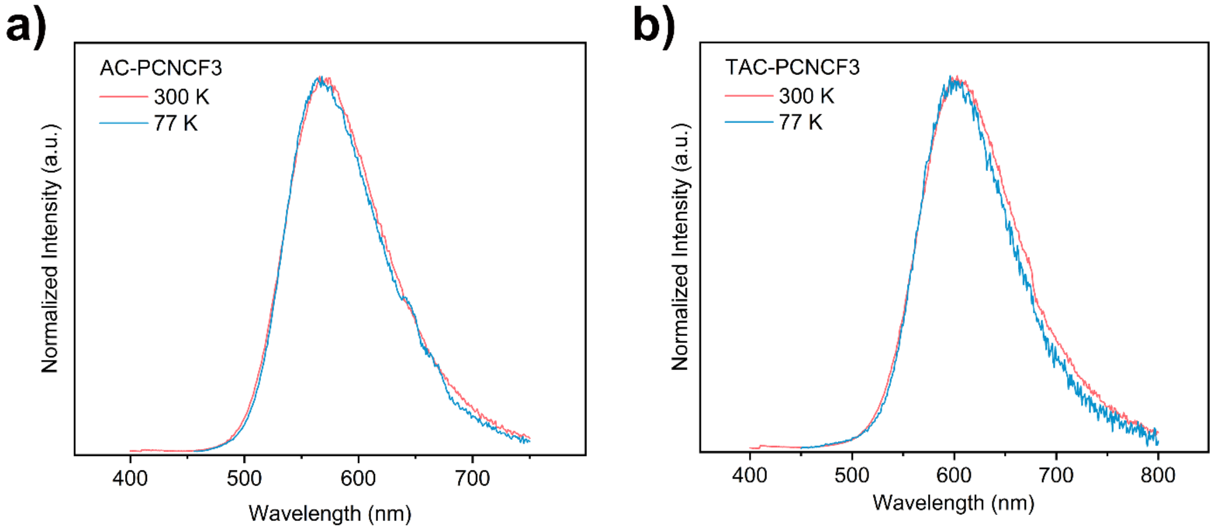

Figure S10. PL spectra of a) AC-PCNCF3 in vacuum-deposited 5 wt%-doped mCBP host films and b) TAC-PCNCF3 in vacuum-deposited 5 wt%-doped CBP host films at 300 K and 77 K.

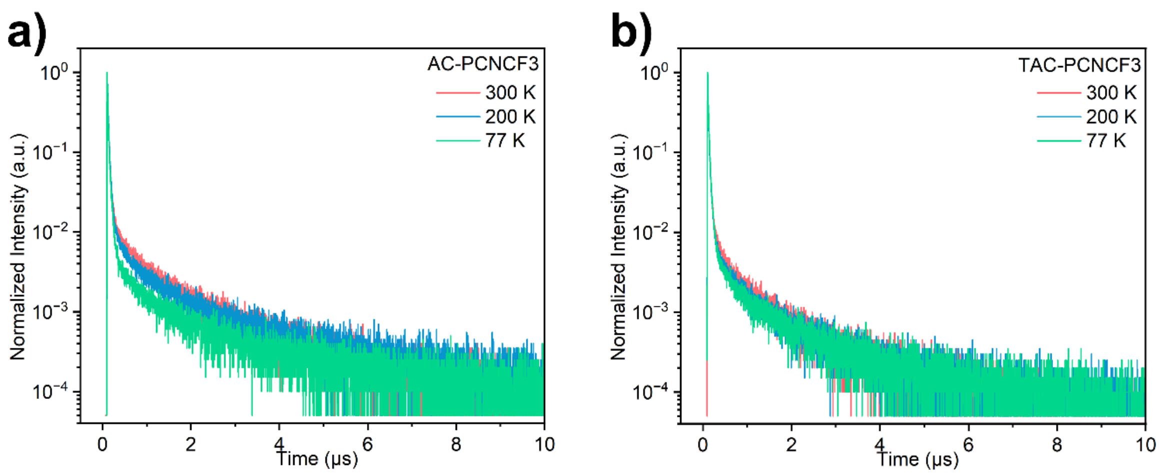

Figure S11. Temperature-dependent transient PL characteristics of a) AC-PCNCF3 in vacuum-deposited 5 wt%-doped mCBP host films and b) TAC-PCNCF3 in vacuum-deposited 5 wt%-doped mCBP host films.

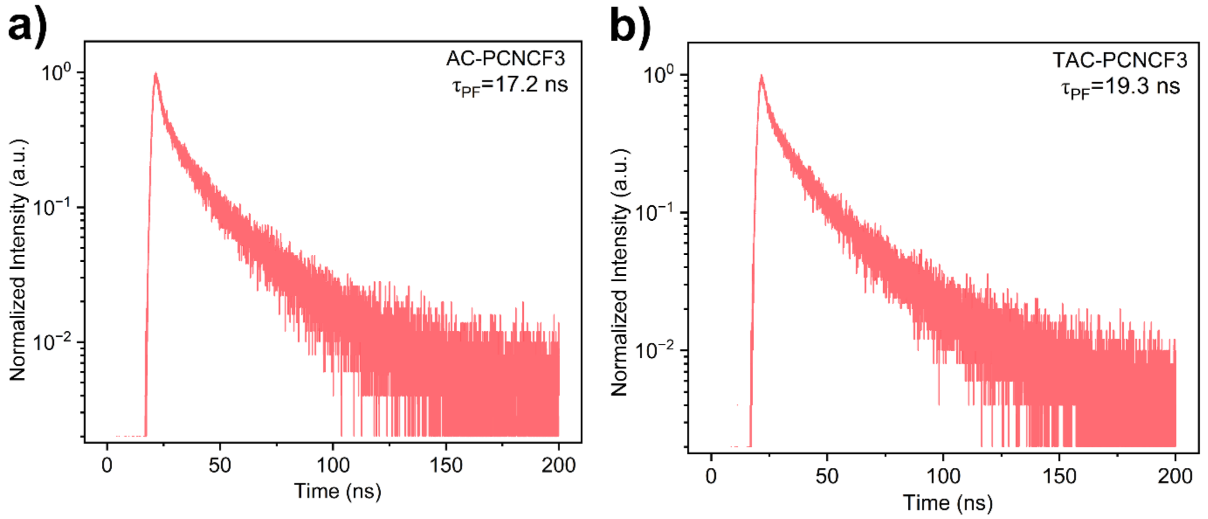

Figure S12. Transient PL decay curves of a)AC-PCNCF3 and b)TAC-PCNCF3 in vacuum-deposited 5 wt%-doped mCBP host films at 300K. The figure shows the prompt component at 300 K.

Estimation for the Photophysical parameters

Rate constants of the investigated compounds in 5 wt% mCBP films at room temperature are determined from the measured quantum yields and lifetimes of the prompt fluorescence (PF)

and delayed fluorescence (DF) components according to equations S1-S6[4].

$$k_{PF} = \frac{1}{\tau_{PF}}$$ Equation S1

$$k_{DF} = \frac{1}{\tau_{DF}}$$ Equation S2

$$k_{RISC} = \frac{k_{PF}+k_{DF}}{2} - \sqrt{(\frac{k_{PF}+k_{DF}}{2})^2 - k_{PF}k_{DF}(1+\frac{\Phi_{DF}}{\Phi_{PF}})}$$ Equation S3

$$k_{ISC} = \frac{k_{PF}k_{DF}\Phi_{DF}}{k_{RISC}\Phi_{PF}}$$ Equation S4

$$k_r^S \approx \frac{k_{PF}k_{DF}}{k_{RISC}}\Phi_{PL}$$ Equation S5

$$k_{nr}^s \approx \frac{k_{PF}k_{DF}}{k_{RISC}}(1-\Phi_{PL})$$ Equation S6

8. Theoretical Calculations

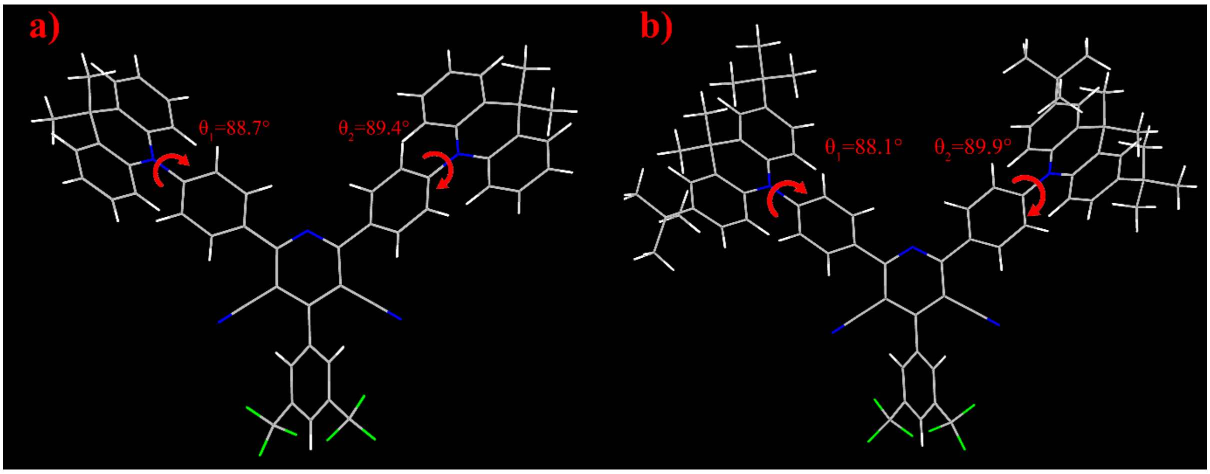

Figure S13. Optimized structures for a) AC-PCNCF3 and b) TAC-PCNCF3

Table S6 Percentage of CT and LE characteristics of different states for AC-PCNCF3 (left) and TAC-PCNCF3 (right).

State	CT (%)	LE (%)	CT (%)	LE (%)
S_1	93.579	6.421	93.806	6.194
T_1	93.581	6.419	93.807	6.193
T_2	93.576	6.424	93.606	6.394
T_3	1.363	98.637	94.832	5.168
T_4	94.656	5.344	94.857	5.143
T_5	94.645	5.355	3.695	96.305

9. Device Fabrication

Glass substrates pre-coated with a 120 nm of indium tin oxide (ITO) with a sheet resistance of 15 Ω per square were successively cleaned in ultrasonic bath of deionized water, acetone, and isopropanol for 15 minutes. Then ITO glass substrates were dried by a steam of Ar_2 and treated by UV-ozone for 15 minutes. The organic materials were deposited onto the ITO-coated substrates at a rate of 1 Å s^{-1}under high vacuum (<2×10^{-5}) by thermal evaporation in vacuum chamber. Then LiF and Al were successively deposited at a rate of 0.1 Å s^{-1} and 5 Å s^{-1}, respectively. The electroluminescence (EL) spectra and Commission Internatinale de L'Eclairage coordinates (CIE) of the OLEDs were recorded by an integrated optoelectronic performance test system with a calibrated spectra radiometer (TOPCON SR-UL1R). The current efficiency and power efficiency were measured using Keithley 2400 source meter. All the measurements were carried out in a nitrogen-filled glove box at room temperature.

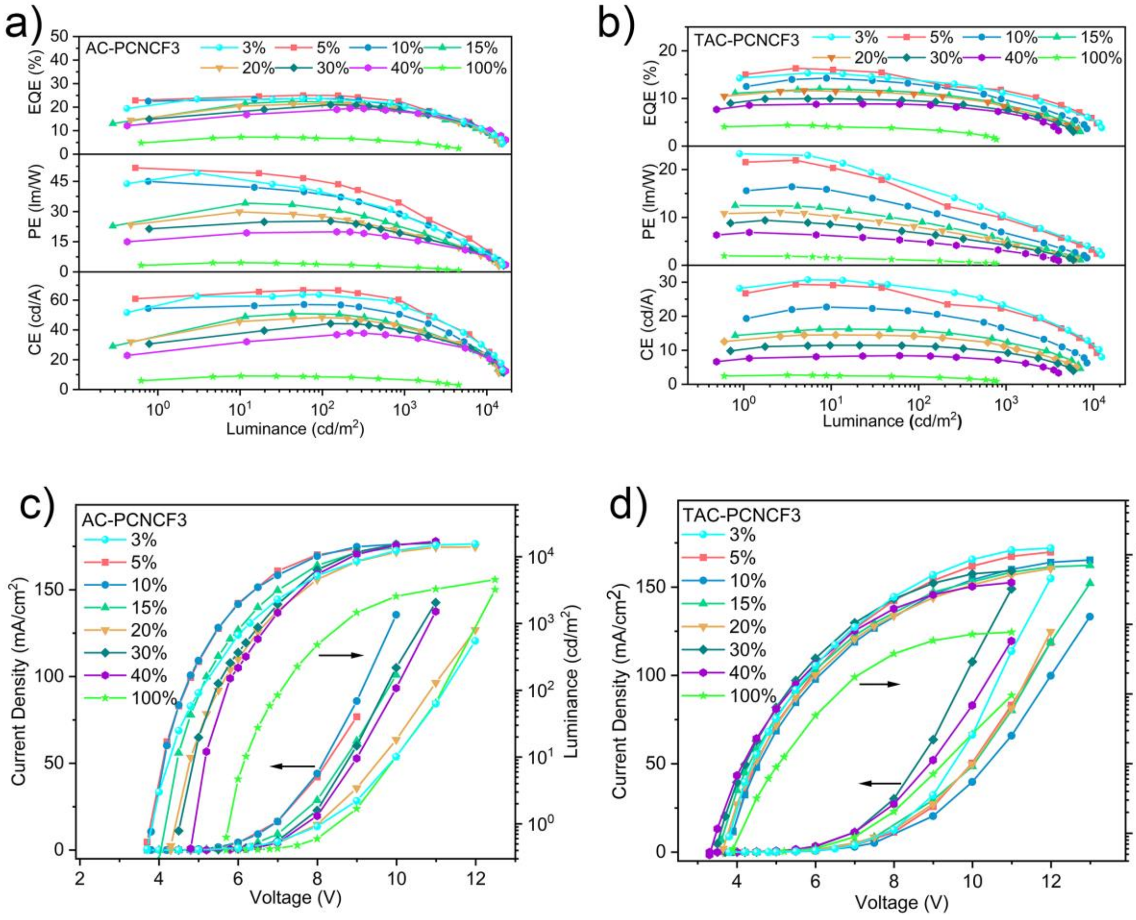

Figure S14. Current efficiencies (CE), power efficiencies (PE) and external quantum efficiencies (EQE) of a) AC-PCNCF3 and b) TAC-PCNCF3 based OLEDs at different doping concentrations; Current density-luminance-voltage (J-L-V) characteristics of c) AC-PCNCF3 and d) TAC-PCNCF3 based OLEDs at different doping concentrations.

Table S7. Summary of device performances of AC-PCNCF3 and TAC-PCNCF3 in different concentration doped mCBP host matrix. (Part I)

Emissive Layer	Concentration	V_{on} (V)	L_{max} (cd/m^2)	λ_{EL} (nm)[a)]	CIE (x,y)[a)]
	3%	3.8	15550	578	(0.49, 0.49)
	5%	3.8	13280	581	(0.50,0.49)
	10%	3.9	15410	586	(0.52,0.47)
	15%	4.1	13680	591	(0.54,0.47)
AC-PCNCF3	20%	4.4	14050	594	(0.54,0.45)
	30%	4.6	16020	595	(0.55,0.44)
	40%	4.9	17020	600	(0.57,0.43)
	100%	5.8	4546	626	(0.61,0.38)
	3%	3.9	11540	601	(0.55,0.44)
	5%	4.0	10920	603	(0.56,0.43)
	10%	3.9	8398	612	(0.58,0.42)
	15%	3.7	7034	622	(0.60,0.40)
TAC-PCNCF3	20%	3.7	6324	625	(0.61,0.39)
	30%	3.6	5876	626	(0.62,0.38)
	40%	3.5	3977	635	(0.64,0.36)
	100%	4.2	774	653	(0.66,0.34)

[a)] λ_{EL} and CIE recorded at 6V.

Table S8. Summary of device performances of AC-PCNCF3 and TAC-PCNCF3 in different concentration doped mCBP host matrix. (Part II)

Emissive Layer	Concentration	CE (cd/A)[a)]	PE (lm/W)[a)]	EQE (%)[a)]	Roll-off (%)[b)]
AC-PCNCF3	3%	63.7/63.5/55.4	49.2/39.5/27.6	23.7/23.6/20.3	14%
	5%	66.7/66.6/57.9	51.7/45.2/32.9	25.0/24.9/22.2	11%
	10%	57.0/57.1/49.4	45.0/38.4/27.4	23.7/23.5/20.4	14%
	15%	51.0/50.7/43.0	34.0/31.5/21.7	22.4/22.3/18.4	18%
	20%	48.3/47.7/41.5	29.9/27.6/19.6	21.5/21.5/18.4	14%
	30%	44.2/43.2/39.2	25.3/25.3/18.4	21.0/20.5/18.3	13%
	40%	37.9/35.9/35.1	19.9/20.0/16.4	19.5/18.3/17.9	8%
	100%	9.1/8.5/6.4	4.6/3.7/2.4	7.3/6.8/5.3	27%
TAC-PCNCF3	3%	30.5/28.1/22.8	23.2/16.3/10.2	15.3/13.7/11.1	27%
	5%	29.3/25.6/21.8	21.9/14.7/9.6	16.3/13.8/11.6	29%
	10%	22.7/21.2/16.1	16.4/11.6/6.7	14.3/12.9/9.6	33%
	15%	16.2/15.8/12.4	12.5/9.0/5.1	12.0/11.3/8.5	29%
	20%	14.5/14.0/11.0	11.1/7.9/4.5	11.6/10.7/8.2	29%
	30%	11.5/11.3/9.3	9.4/6.7/4.1	10.0/9.5/7.5	25%
	40%	8.4/8.3/6.8	6.9/4.9/3.0	8.8/8.7/7.0	20%
	100%	4.4/2.2/	2.0/1.0/	4.4/3.5/	

[a)] The efficiency values of maximum/at 100 cd/m^2/at 1000 cd/m^2; [b)] Efficiency roll-off calculated at 1000 cd/m^2.

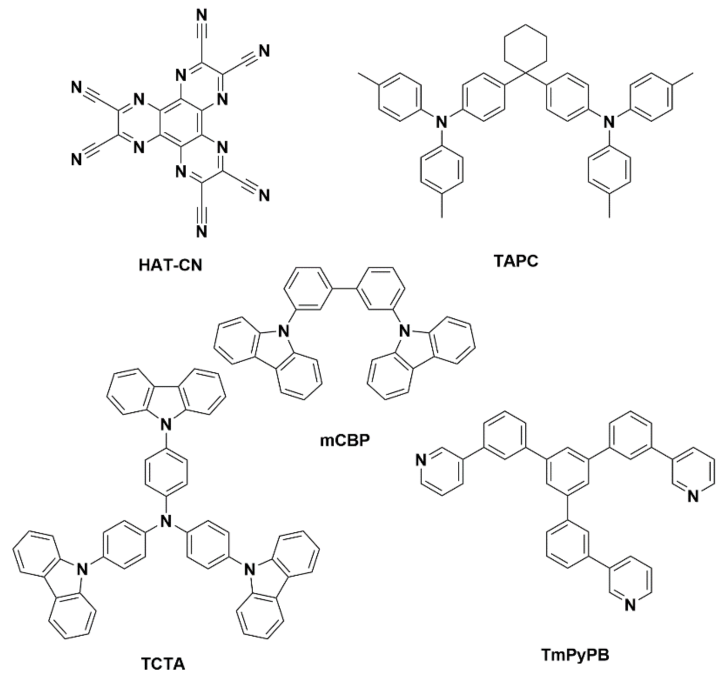

Figure S15. The Molecular structures of materials used in OLEDs.

10. Reference

[1] V. Chhiba, M. L. Bode, K. Mathiba, W. Kwezi, D. Brady, *J. Mol. Catal. B: Enzym.* **2012**, 76, 68.

[2] R. Huang, N. A. Kukhta, J. S. Ward, A. Danos, A. S. Batsanov, M. R. Bryce, F. B. Dias, *J. Mater. Chem. C* **2019**, 7, 13224.

[3] T.-L. Wu, M.-J. Huang, C.-C. Lin, P.-Y. Huang, T.-Y. Chou, R.-W. Chen-Cheng, H.-W. Lin, R.-S. Liu, C.-H. Cheng, *Nat. Photonics* **2018**, 12, 235.

[4] a) Y. Wada, H. Nakagawa, S. Matsumoto, Y. Wakisaka, H. Kaji, *Nature Photonics* **2020**, 14, 643; b) X. L. Chen, X. D. Tao, Z. Wei, L. Meng, F. L. Lin, D. H. Zhang, Y. Y. Jing, C. Z. Lu, *ACS Appl. Mater. Interfaces* **2021**, 13, 46909.
